# PSA Dynamics, Bounce, and Biochemical Recurrence Definitions After Low-Dose-Rate Brachytherapy for Prostate Cancer: A Narrative Review with Cross-Modality Comparison

**DOI:** 10.3390/diagnostics16213689

**Published:** 2026-11-08

**Authors:** Nikolay Kahchiev, Elenko Popov, Vaska Vasileva, George Dimitrov

**Affiliations:** 1 Department of Radiotherapy, University Hospital “Tsaritsa Yoanna”, Medical University of Sofia, 1527 Sofia, Bulgaria; 2 Department of Urology, University Hospital “Tsaritsa Yoanna”, Medical University of Sofia, 1527 Sofia, Bulgaria; 3 Department of Medical Oncology, University Hospital “Tsaritsa Yoanna”, Medical University of Sofia, 1527 Sofia, Bulgaria; 4 Centre of Competence in Personalized Medicine, 3D and Telemedicine, Robotic Assisted and Minimally Invasive Surgery—Leonardo da Vinci, 5800 Pleven, Bulgaria

**Keywords:** prostate cancer, low-dose-rate brachytherapy, permanent seed implantation, prostate-specific antigen, prostate-specific antigen dynamics, PSA nadir, PSA bounce, biochemical recurrence, Phoenix criteria

## Abstract

**Background:** Prostate-specific antigen (PSA) dynamics provide the earliest indicator of treatment outcome. Understanding them, including nadir kinetics and PSA bounce, is essential for refining biochemical recurrence (BCR) definitions and guiding clinical decision making. **Methods:** A literature search was conducted across the PubMed, Scopus, and Web of Science databases. Backward citation chaining of selected references resulted in a final synthesis of 143 studies. Iodine-125 constitutes the primary radioisotope across the reviewed literature. **Key Findings:** Following low-dose-rate brachytherapy (LDR-BT), a PSA nadir < 0.2 ng/mL is strongly associated with long-term biochemical control. PSA bounce (defined as a rise ≥ 0.2 ng/mL above the current nadir with spontaneous resolution) occurs in approximately 30% to 50% of LDR-BT cohorts, with a median onset of approximately 1.5 years, a median duration of approximately 1 year, and a median amplitude < 1 ng/mL. Up to 18% of bounces exceed 2.0 ng/mL, accounting for nearly half of all early rises triggering the Phoenix criteria. Superior dosimetric parameters are associated with longer time to nadir and lower nadir values. Within LDR-BT cohorts, younger patient age is the most consistent predictor of bounce incidence, whereas between-study variation is primarily driven by differences in treatment modality. LDR-BT and hypofractionated regimens report higher bounce rates than those for conventional external beam radiotherapy, although unadjusted comparisons are confounded. Onset timing is the most useful feature distinguishing between bounce and BCR. **Conclusions:** Static, single cutoff BCR definitions post-LDR-BT may be suboptimal for surveillance. This observational synthesis suggests a hypothesis-generating, time-windowed approach (Phoenix definition during early follow-up (< 4 years) and an absolute threshold of PSA > 0.2 ng/mL thereafter) that requires prospective validation and is not a recommendation for clinical use.

## 1. Introduction

Prostate cancer (PC) is the second most commonly diagnosed cancer in men worldwide, with an estimated 1.5 million new cases and approximately 397,000 deaths in 2022 [1]. As a result of population growth and aging, the global burden is projected to rise substantially over the coming decades, with modelling studies anticipating a near-doubling of cases by 2040–2050 [2]. Radical prostatectomy (RP) and radiotherapy (RT) are both standard options for the definitive treatment of non-metastatic disease and offer comparable long-term cancer-specific survival [3]. Among RT modalities, low-dose-rate (LDR) prostate brachytherapy (BT) is delivered in a single procedure and provides durable biochemical control for appropriately selected low- and intermediate-risk patients. Its toxicity profile is generally acceptable, and quality-of-life outcomes are favourable in the long term; however, LDR-BT is associated with transient acute urinary irritative and obstructive symptoms that typically peak within the first three months and resolve over 6–12 months, and it carries higher early genitourinary toxicity than that of stereotactic, high-dose-rate, or image-guided external-beam approaches [4]. These considerations, together with its single-session delivery and, in some healthcare systems, comparatively low cost, make LDR-BT an attractive option as the incidence of PC continues to increase.

The introduction and widespread adoption of prostate-specific antigen (PSA) testing in the late 1980s substantially improved disease monitoring, enabling detection of recurrence long before radiological or clinical signs appear. This lead time necessitates a biomarker-based definition—biochemical recurrence (BCR)—using a PSA threshold or derived kinetic parameter that, once crossed, indicates the cancer is considered to have recurred, distinct from true clinical recurrence. Histological confirmation is of limited value in this window: post-radiation biopsies performed earlier than 2–3 years after treatment are difficult to interpret because of persistent treatment effects [5], and biopsy will also fail to identify disease when only extraprostatic micrometastases are present. BCR, therefore, carries intrinsic uncertainty, with a meaningful false-positive rate and, less commonly, false-negative results. Optimising the predictive value of BCR for clinical recurrence requires selecting the most appropriate definition for the specific clinical context while accounting for confounding factors and PSA dynamics that can interfere with it. Understanding early PSA dynamics allows timely salvage intervention for localised recurrence or early systemic escalation for patients at high risk of distant metastasis.

Despite the distinct radiobiology and PSA behaviour of LDR-BT, BCR is still most often assessed using definitions developed for external-beam radiotherapy (EBRT) or RP. This review therefore aims to (1) characterise absolute PSA decline, time to nadir, and nadir depth after LDR-BT; (2) define the incidence, timing, duration, magnitude, and prognostic meaning of PSA bounce; (3) compare these parameters across radiotherapy modalities; and (4) critically appraise BCR definitions and propose directions for a time-adapted framework. Given the observational nature of the underlying evidence, the proposed framework is intended to be hypothesis-generating rather than a substitute for current guideline-endorsed BCR definitions. This review is distinguished from prior syntheses in three respects. First, whereas earlier narrative work addressed the PSA bounce phenomenon in isolation [6], and subsequent meta-analyses each quantified a single dimension of it—prognostic value [7,8] or descriptive characteristics [9]—the present review integrates absolute PSA decline, time to and depth of nadir, decline velocity, bounce incidence/timing/duration/magnitude, and the comparative performance of competing BCR definitions within one frame. Second, it is explicitly anchored to LDR and to the ablative, near-surgical nadir that distinguishes it from EBRT, rather than treating all radiation modalities as interchangeable; cross-modality data are used as comparators to isolate what is specific to permanent-seed implantation. Third, it translates these observations into a time-windowed surveillance approach—applying a nadir-referenced (Phoenix) criterion during the early, bounce-prone window and transitioning to an absolute low-PSA threshold thereafter—presented as a hypothesis-generating framework requiring prospective validation.

## 2. Methods

This is a narrative review; a systematic protocol was not registered, and no formal risk-of-bias or quality appraisal was undertaken. To identify relevant literature, a structured search was conducted across PubMed, Scopus, and Web of Science for articles published between January 2010 and June 2026. The January 2010 cutoff applied to the database search only, and earlier seminal papers were incorporated separately through citation chaining (see below). Queries were restricted to title, abstract, and keyword fields. Database-specific algorithms combined the following terms with Boolean operators: (“prostate cancer”) AND (“low risk” OR “intermediate risk” OR “favourable risk” OR “unfavourable intermediate risk”) AND (“LDR brachytherapy” OR “low dose rate brachytherapy” OR “low-dose-rate brachytherapy” OR “LDR-BT” OR “LDR BT” OR “seed implantation” OR “permanent seed implant”) AND (“PSA” OR “prostate-specific antigen”). After retrieval, duplicate records were removed, and all unique records were screened independently by two reviewers against the title and abstract, with full-text assessment of potentially eligible records and disagreements resolved by discussion. Searches were executed on 3 June 2026, restricted to English-language publications, with no restriction on publication type at the search stage. Eligibility required original or synthesized data on post-treatment PSA kinetics (nadir, time to nadir, decline velocity, bounce, or a biochemical-recurrence endpoint) in patients treated with definitive radiotherapy for localized prostate cancer. Records were excluded for the following reasons: reports in a non-English language; single-case reports; cohorts of fewer than 20 patients; absence of relevant PSA-kinetic or recurrence data; and substantial data-quality limitations precluding interpretation. Fifty-three primary articles met these criteria through the database search. Because the a priori strategy was oriented toward LDR-BT, low-, and intermediate-risk disease, backward citation chaining of the selected references captured foundational studies, cross-modality comparisons, high-risk and mixed-risk cohorts, relevant systematic reviews and meta-analyses, consensus and practice-parameter statements, and pre-2010 literature. This added 90 publications, for a final corpus of 143. The corpus comprises both primary clinical studies and secondary, meta-analytic, and consensus publications. The full database-specific search strings and the study-selection flow are provided in the Supplementary File S1. As a narrative review relying substantially on citation chaining (90 of 143 publications, 63%), the corpus may over-represent frequently cited or seminal work and does not constitute an exhaustive, reproducible sample. Exclusion of non-English language publications introduces potential language bias. Consistent with a narrative rather than a systematic review design, a formal quantitative risk-of-bias instrument was not applied, and no quantitative meta-analysis was performed. Studies were nonetheless appraised qualitatively for methodological features known to bias PSA-kinetic endpoints, including retrospective single-institution designs, non-uniform PSA sampling schedules, variable follow-up durations, differing risk-group composition, asymmetric androgen-deprivation therapy (ADT) exposure, and inconsistent definitions of PSA bounce and BCR. Heterogeneity across PSA assay methods, sampling intervals, primary treatment modalities, androgen deprivation therapy exposure, and diagnostic criteria limits direct comparison of reported outcomes; these sources of variation are the explicit reason why effect estimates are reported as comparative ranges across studies rather than as pooled quantitative metrics. Unless otherwise specified, ‘LDR-BT’ in this review denotes permanent iodine-125 (^125^I) seed implantation, the predominant isotope in the corpus; palladium-103 (^103^Pd) and cesium-131 differ in half-life and initial dose rate and, where reported, are identified explicitly. High-dose-rate brachytherapy (HDR-BT), EBRT (conventionally fractionated and hypofractionated), stereotactic body radiotherapy (SBRT), and combined EBRT + BT approaches are treated as distinct comparators and are not pooled with permanent-seed ^125^I data.

## 3. Narrative Synthesis

### 3.1. Post-Treatment PSA Dynamics

#### 3.1.1. Absolute PSA Decline

After radiotherapy, PSA declines gradually and continuously, in contrast to its rapid fall to undetectable levels after RP, which reflects clearance based on the serum half-life of PSA [10,11]. The PSA nadir (nPSA)—the lowest value recorded after definitive treatment—is therefore reached far later after LDR-BT than after surgery. Crook et al. illustrated this protracted decline: the median PSA after ^125^I LDR-BT without ADT was 0.3, 0.18, 0.09, and <0.05 ng/mL at 30, 36, 48, and 60–78 months, respectively [12]. Other series report a last recorded PSA of 0.01–0.14 ng/mL, depending on follow-up duration [12,13,14,15]. The same time dependence was shown in 1569 patients treated with LDR-BT, with or without EBRT, and stratified by nPSA (<0.1, 0.1–0.2, 0.2–0.5, >0.5 ng/mL): the distribution shifted markedly toward lower categories over time, from 19.1%/22.8%/31.4%/26.7% at 3 years to 49.9%/25.2%/18.0%/6.9% at 5 years [16]. PSA continues to fall for years after LDR-BT; with sufficient follow-up (often 8–10 years), the proportion of patients reaching very low nadirs continues to increase, and absolute PSA and progression-free curves converge. In long-term series, PSA remains stable at a surgical-ablation level (nadir ~ 0.03 ng/mL) in roughly 86% of patients. These findings are consistent with the high, ablative-range biologically effective dose (BED) delivered by LDR-BT. To enable cross-cohort comparison and assess the consistency of reported post-treatment PSA kinetics, the included clinical series are extracted and summarized in Supplementary Table S1.

#### 3.1.2. Time to PSA Nadir

The post-radiotherapy PSA decline is biphasic across all modalities. An initial phase of relatively rapid decline over roughly the first 1–2 years is followed by a slower, more gradual phase. The prevailing interpretation attributes the first phase to the rapid death of radiosensitive malignant cells and the second to gradual ablation of the benign prostatic epithelium, which has a longer cell-cycle time and lower radiosensitivity [17,18,19]; the precise cellular basis remains inferential. The second phase continues to approximately year 5, e.g., Logghe et al. reported that 82% of patients had reached nadir by the end of year 5 [20]. For ^125^I LDR-BT, the reported median time to nadir is approximately 30–56 months (2.5–4.7 years), and continued PSA decay beyond 4 years itself predicts durable biochemical control [12,14,17,21,22]. Beyond the conventional two-phase model, an additional ultra-slow decline in a subset of patients—reaching an even lower true nadir at extended follow-up—is plausible and consistent with long-term LDR-BT surveillance data, though it has not been formally characterised as a distinct kinetic phase. Regarding the time to fulfil the surgical biochemical-control threshold (PSA < 0.2 ng/mL), Soyano et al. reported a median of 4 years, and Chéchile Toniolo observed that 71.7% and 86.8% of patients reached it by 4 and 5 years, respectively [11,23].

#### 3.1.3. Factors Affecting Time to PSA Nadir

Several patient-, tumour-, and treatment-related factors modulate the time to nadir. PSA bounce is among the most consistent. Bounce prolongs the time to nadir, reflecting its protracted natural history [12], and patients who bounce have higher median PSA levels at 6 weeks and 1 year than do non-bouncers [24,25]. A large multimodality meta-analysis identified PSA bounce as the most consistent clinical factor prolonging time to nadir across all RT modalities [9].

ADT strongly confounds early PSA kinetics. By suppressing androgen-dependent PSA production, ADT drives PSA to very low levels far faster than does radiation alone—in combined RT + ADT cohorts, 85–90% of patients reach PSA ≤ 0.1 ng/mL within 6–12 months, vs. only 23–36% after RT alone—so the early “nadir” during ADT is partly pharmacologic rather than a measure of tissue ablation [26]. Ohashi et al. illustrate this: a treatment-heterogeneous cohort reached an nPSA of 0.08 ng/mL, whereas the hormone-naive subgroup reached 0.14 ng/mL [27]. After ADT cessation, testosterone recovery is slow and variable—non-castrate levels are typically regained within 6–15 months, but full eugonadal recovery often takes years and is influenced by ADT duration, patient age, baseline testosterone, and agent class—and the accompanying testosterone-driven PSA rise can be misread as a bounce or as early recurrence [28,29]. Accounting for ADT status and, where available, serum testosterone is therefore essential when interpreting nadir kinetics.

The relationship between tumour burden and time to nadir is inconsistent and likely confounded. In 2218 patients treated with LDR-BT ± EBRT, Soyano et al. found that longer time to PSA < 0.2 ng/mL was associated with younger age, larger prostate volume, higher initial PSA (iPSA), and LDR-BT monotherapy; within the monotherapy subgroup, day-30 volume receiving 200% of the prescription dose (V200) and neoadjuvant ADT duration were additional predictors [11]. Conversely, a comparative study of LDR-BT, conventionally fractionated EBRT (CF-EBRT), and hypo-fractionated EBRT (H-EBRT) found that a higher percentage of positive biopsy cores correlated weakly with a marginally faster time to nadir [22], and the multimodality meta-analysis found no association between risk group and time to nadir, although these results require caution given the confounding introduced by differing radiation radiobiology and asymmetric hormone therapy use [9]. These divergent findings probably reflect confounding—higher iPSA in Soyano et al. co-varies with larger prostate volume and monotherapy selection, whereas positive-core burden is a within-modality signal—rather than a single monotonic effect of tumour burden.

Treatment factors are more reproducible. LDR-BT, alone or combined with EBRT, is consistently associated with a longer time to nadir than is EBRT alone [30]. Intraoperative LDR-BT planning yields both a shorter time to nadir and lower nadir values than does preoperative planning, attributed by the authors to superior dosimetric coverage [31]. A dose effect is also seen with EBRT, where doses > 70 gray (Gy) prolong time to nadir; in the large multi-institutional analysis by Ray et al. (4833 patients), a dose ≥ 70 Gy was independently associated with a lower nPSA and a longer time to nadir across all risk groups [32,33], and SBRT dose-response data show steeper PSA decay and lower nadirs with dose escalation [34].

Higher BED and better dosimetric coverage are thought to increase ablation of the benign prostatic epithelium; because these cells turn over slowly, the resulting PSA decline manifests gradually, producing both a longer time to nadir and a lower absolute nadir. Younger age and PSA bounce are partially correlated, and their effect has increasingly been linked to immune-mediated processes—though this remains one of several proposed mechanisms (radiation prostatitis, benign-tissue inflammation, and microvascular injury among them)—rather than an established cause [35,36].

#### 3.1.4. Prognostic Significance of Time to PSA Nadir

The prognostic meaning of the time to nadir is best understood in conjunction with nadir depth rather than in isolation. Patients whose PSA declines slowly and continuously tend to reach a lower absolute nadir than those whose PSA falls quickly and then plateaus [20] and because a lower nadir is one of the strongest predictors of biochemical control, a gradual, sustained decline is generally favourable. In the multi-institutional analysis by Ray et al. (4833 patients), a shorter time to nadir was associated with worse biochemical and distant-metastasis-free survival across all risk categories, and independent of nadir depth, with 8-year PSA disease-free survival rising from 27% for a time to nadir < 6 months to 75% for ≥24 months [32]. Similarly, Vicini et al. found that both nadir and time to nadir had the strongest association with 10-year biochemical control in a cohort of 2100 patients [37]. Throughout this review, ‘cure’, where used, follows the terminology of the cited studies and denotes freedom from biochemical failure during follow-up; absolute PSA thresholds are prognostic markers of low recurrence risk and do not constitute proof of cure.

This favourable association is conditional, however, on eventually achieving a deep nadir; a prolonged decline that settles at a high nadir carries the opposite meaning. Accordingly, Taussky et al. found time to nadir prognostic in a mixed EBRT/LDR-BT cohort and in EBRT alone, but not in the LDR-BT subgroup [38]—plausibly because nearly all LDR-BT patients reach a very low nadir, leaving little residual discriminatory power in the timing itself. In another mixed cohort, taking longer than 5 years to reach PSA < 0.5 ng/mL was associated with higher, not lower, BCR rates than was reaching that threshold sooner [39], consistent with a sluggish decline that fails to reach an ablative nadir.

Two caveats temper these observations. First, much of the apparent benefit of a long time to nadir is subject to lead-time bias: in the largest dedicated analysis (2506 RT patients), longer time to nadir independently predicted both biochemical-recurrence-free survival and overall survival, but after correction for lead-time bias, the association persisted only for biochemical-recurrence-free survival, not overall survival [40]. Second, the interval to nadir is itself a downstream product of the multifactorial determinants discussed above, so it should be interpreted as a composite marker rather than as an independent biological driver.

Mechanistically, a protracted, sustained PSA decline plausibly reflects progressive ablation of both malignant and benign tissue, and the resulting deep nadir is associated with a lower likelihood of residual viable cancer. Conversely, when PSA clearance is abnormally sluggish—reaching only modestly depressed levels over a timeline exceeding the reported median time to nadir—the active tumour signal may be masked by the slow attrition of benign epithelium, leaving a protracted, shallow decline as an early indicator of occult malignant persistence. The discriminating feature, therefore, is not the duration of decline per se but whether that decline ultimately arrives at an ablative nadir.

#### 3.1.5. Inter-Modality Differences in Time to PSA Nadir

In between-study comparisons, ^125^I LDR-BT monotherapy without ADT has most often been reported to have the longest time to nadir among radiotherapy modalities. It exceeds that of combined LDR-BT plus EBRT, EBRT monotherapy (CF-EBRT or H-EBRT), high-dose-rate brachytherapy (HDR-BT) monotherapy, and conformal proton-beam boost after photon EBRT [11,17,21,22,41]. HDR-BT and SBRT occupy an intermediate position: in the multi-institutional analysis of 3502 patients, LDR-BT reached both the deepest nadir and the most protracted decay pattern relative to those of HDR-BT and SBRT [17], and when Kishan et al. compared SBRT, HDR-BT, and 78–81 Gy intensity-modulated radiotherapy (IMRT), the median time to nadir was approximately 35, 34, and 21 months, respectively [42]. In these studies, conventionally fractionated EBRT had the shortest latency to nadir, and even at dose-escalated levels of 78–81 Gy, it did not approach the latency reported for LDR-BT.

This ranking is plausibly driven by radiation delivery kinetics. LDR-BT delivers its dose continuously at a low dose rate over the months-long decay of the implanted isotope, in contrast to the high-dose-rate, temporally compressed delivery of HDR-BT, SBRT, and EBRT. Consistent with a dose-rate effect, ^103^Pd—which has a shorter half-life (~17 days) and higher initial dose rate than those of ^125^I (~60 days)—produces a faster PSA response and a shorter time to nadir [43]; a recent international multi-institutional study also reported superior biochemical control with ^103^Pd over ^125^I, though time to nadir was not disclosed [44], suggesting that isotope dose-rate may influence both kinetics and outcome.

Reports on boost regimens are broadly concordant: one series found a slightly longer time to nadir for HDR-BT boost than for LDR-BT boost [45].

Overall, the available evidence is consistent with ^125^I LDR-BT monotherapy without ADT, having the longest time to nadir of the radiotherapy modalities, with HDR-BT and SBRT achieving intermediate times and CF-EBRT the shortest. However, these rankings derive largely from comparisons between cohorts that differ in age, risk group, ADT use, dose, follow-up and PSA sampling, with limited matching or formal adjustment, and head-to-head comparisons against every modality are lacking. They should be read as descriptive, hypothesis-generating observations and not as established modality effects. Absolute medians also vary considerably across series and dose levels. Importantly, a longer time to nadir should be interpreted cautiously as a between-modality descriptor rather than a within-LDR-BT prognostic marker: as noted above, time to nadir loses prognostic power within LDR-BT cohorts because nearly all patients ultimately reach a very low nadir [38].

#### 3.1.6. PSA Decline Velocity

Post-treatment PSA decline velocity is reported less often than nadir depth, and its prognostic value depends heavily on which phase of decline is measured and whether ADT was used. The early decay rate appears to carry limited independent prognostic weight: historical exponential-kinetics analyses found the post-radiation PSA half-life (~1.9 months to ~2 months) to be uncorrelated with stage, grade, baseline PSA, or subsequent relapse [46,47], and after brachytherapy, the early decline rate has generally not predicted BCR [48]. Ko et al. found no difference in decline velocity by BED [39], and in a comparative analysis, LDR-BT showed a faster early exponential decline than that of EBRT, without any relationship between that velocity and BCR [22].

This apparent lack of prognostic value is specific to the early decay rate, however, and does not extend to the long-term PSA slope or to ADT-treated cohorts, where several large datasets show a clear signal. Among 2566 intermediate- and high-risk patients treated with RT plus ADT, an early PSA reduction ≥ 95% relative to baseline was associated with a significantly lower risk of biochemical relapse and distant metastasis [49], and a post-radiation decline rate ≥ 90% independently predicted BCR in a three-dimensional conformal radiotherapy/ADT cohort [37]. Cavanaugh et al. similarly showed that early PSA kinetics, assessed with a time-and-threshold model, predicted biochemical recurrence-free survival (bRFS) as early as 90 days after EBRT [50]. The prognostic content of PSA kinetics therefore resides principally in the trajectory over time—and in the depth ultimately reached—rather than in the raw early velocity.

Across modalities, the shape of the decay curve differs even when early rates converge. In a comparison of SBRT, HDR-BT, and conventionally fractionated IMRT, the three produced similar decay rates within the first ~1000 days; thereafter, PSA continued to decline (more slowly) after SBRT and HDR-BT but plateaued after IMRT [42]. Ablative therapies show a latent early response followed by faster decay in the 6–24 month window [41], and SBRT characteristically produces a steep initial decline that slows over time to a lower nadir [51]. In a combined LDR-BT/HDR-BT/SBRT cohort, all modalities declined rapidly for two years and then more gradually thereafter; SBRT had the slowest late decline, while LDR-BT decayed at a moderate late pace before flattening around year 5 [17]. A study of HDR-BT, SBRT, and CF-EBRT found comparable velocity at 33 months, after which SBRT- and HDR-BT-treated patients continued to show a decline [42].

In summary, the early (first 1–2 year) PSA decline rate is a weak and inconsistent predictor of BCR after brachytherapy and is largely independent of BED and modality. The prognostically meaningful kinetic signals are the later PSA slope and the eventual nadir depth; the differences in late decline velocity across modalities principally reflect lower-BED treatments reaching their (higher) nadir sooner, whereas ablative treatments continue a slow decay toward a deeper nadir.

#### 3.1.7. Prognostic Significance of PSA Nadir Depth

A lower absolute PSA nadir is one of the most consistent predictors of long-term biochemical control after LDR-BT, with an almost linear relationship between nadir depth and biochemical relapse-free survival [20,28,29,35,52,53,54]. In the multi-institutional analysis by Kuban et al. (2693 patients), 8-year PSA relapse-free survival fell stepwise across nadir strata—92%, 86%, 79%, and 67% for nadir values of 0–0.49, 0.5–0.99, 1.0–1.99, and >2.0 ng/mL, respectively—and the median nadir among patients disease-free at 8 years was 0.1 ng/mL, with 90% achieving <0.6 ng/mL [10]. The same relationship holds across other radiotherapy modalities: EBRT [32,36,55], EBRT combined with LDR- or HDR-BT [56,57], and HDR-BT monotherapy [58]. Nadir depth is therefore a central benchmark of treatment success.

Progressively lower thresholds confer progressively lower risk. A nadir < 0.5 ng/mL was one of the earliest validated thresholds (Grado et al.: 5-year disease-free survival 93% for <0.5 vs. 25% for 0.5–1.0 and 15% for >1.0 ng/mL) [59] and remains prognostic in modern series [19,22,39,60,61]. A nadir < 0.2 ng/mL is a commonly cited threshold associated with markedly lower BCR—roughly a sevenfold difference in failure when it is not reached [22,23,39,48]—and a nadir < 0.1 ng/mL at 5 years renders subsequent BCR exceedingly rare [28,45,62]. Nakai et al. found that in men with normal testosterone, nadirs < 0.1, <0.2, and <0.5 ng/mL were each prognostic for improved clinical-failure-free survival at years 2, 4, and 7 [63].

The prognostic value of nadir depth is critically dependent on testosterone status, and this qualifier should not be treated as incidental. A subcastrate or incompletely recovered testosterone level artificially suppresses PSA, so a low nadir in a hypogonadal or recently ADT-treated patient does not carry the same reassurance as one achieved with eugonadal testosterone. Bitterman et al. found that the prognostic significance of nadir depth for prostate-cancer-specific and all-cause mortality was present only in men with normal testosterone at the time of PSA failure [64], and subcastrate testosterone nadirs are themselves associated with lower measured PSA nadirs and altered outcomes [37]. Contemporary analyses further warn that failure to index PSA to testosterone may cause radiotherapy series to underestimate true BCR rates [29]. Nadir depth should therefore be interpreted alongside a documented testosterone level.

The proportion of patients reaching these thresholds is substantial but risk-group- and ADT-dependent. Across series, nadirs < 0.1 and <0.2 ng/mL are reached by approximately 35–66% and 63–89% of patients, respectively [11,17,21,28,39,41,65]; in the largest ADT-free comparison, 72% reached a nadir < 0.2 ng/mL after LDR-BT, with clear stratification by risk group and modality [44]. Overall, LDR-BT drives roughly three-quarters of patients to a nadir at or below the surgical ablation level (77.1% with 4-year PSA ≤ 0.2 ng/mL in the 14,220-patient pooled analysis) [66], and long-term LDR-BT series confirm durable PSA stability at that level in the large majority (86% stable at a median nadir of 0.03 ng/mL) [30]. This ablative depth, approaching post-prostatectomy values, distinguishes LDR-BT from EBRT and underlies its favourable biochemical outcomes.

#### 3.1.8. Time-Specific PSA Thresholds as Recurrence Predictors

Post-treatment PSA measured at defined time points is prognostic for long-term outcome after LDR-BT, and the strength of that association increases with the follow-up interval at which PSA is assessed. Reported thresholds include a week 4–8 PSA < 2 ng/mL [53], month 3 PSA < 1 ng/mL [67], year 1 PSA < 0.285 ng/mL [26], year 2 PSA < 0.5 ng/mL [68], and year 4 PSA values of <0.2, 0.35, 0.4, and 1 ng/mL [35,52].

The most robust of these is the 4-to-5-year PSA. In the pooled multi-institutional analysis of 14,220 patients (2503 with an EBRT component), a 4-year PSA ≤ 0.2 ng/mL was associated with a freedom-from-recurrence of 98.7% at 10 years and 96.1% at 15 years, vs. 93.5% and 86.8% for >0.2 to ≤0.5 ng/mL, with progressively worse rates above 0.5 and above 1 ng/mL; the authors proposed a 4-year PSA ≤ 0.2 ng/mL as a biochemical definition of cure after brachytherapy [66]. It should be noted that this is a prognostic association observed in retrospective data and not proof of cure. This threshold has been externally validated (10-year disease-free survival (DFS) at 97.5% for ≤0.2 vs. 41.8% for >1 ng/mL), although the same validation showed materially lower DFS in unfavourable intermediate-risk disease (75.9%), indicating that the prognostic value of this threshold depends on the risk group [69]. Lo et al. added a dynamic early signal within this window: consecutive PSA rises > 0.2 ng/mL from 24→36 and 36→48 months predicted failure rates of 52%, 79%, and 100%, respectively [35].

Contrary to the view that no reliable early marker exists, time-limited PSA within the first post-treatment year has repeatedly proven prognostic when interpreted as a continuous variable and with attention to confounders. In an ADT-free cohort of 663 patients, PSA nadir within 12 months (nPSA12) independently predicted biochemical failure and distant metastasis after both LDR-BT and EBRT—a 7-year biochemical-failure-free survival of 99.1% vs. 90.2% for LDR-BT with nPSA12 ≤ 0.7 vs. > 0.7 ng/mL [70]. Alcántara et al. (1000 patients, RT alone) found nPSA12 to be an independent predictor of distant metastasis, cause-specific and overall mortality (10-year distant-metastasis of 4% vs. 19% for ≤2 vs. >2 ng/mL) [71]. The timing of early PSA failure is similarly prognostic: among 2818 brachytherapy patients, PSA failure within 3 years independently predicted distant metastasis (HR 3.92), as well as prostate-cancer-specific and overall mortality, despite higher BED and longer ADT [72].

Two caveats govern the interpretation of early time points. First, ADT status is decisive. In a population-based study of 5299 men, detectable PSA at 6 and 12 months predicted prostate-cancer-specific mortality only among RT + ADT patients (12-month subhazard ratio 37.9) and not among RT-alone patients because ADT compresses the early decline and unmasks poor responders [73]; thresholds derived from ADT-free brachytherapy cohorts therefore cannot be applied to ADT-treated patients, or vice versa. Second, in the very early window, PSA bounce remains prevalent and can obscure the signal, which likely underlies discordant early-timepoint findings such as those of Thomas et al., who found year 1 and 2 PSA to be non-prognostic [54].

Reaching a low PSA at year 4 or later is thus a robust independent favourable factor, aided by the near-complete resolution of PSA bounce by that time. Even so, biochemical recurrence continues to accrue beyond 4 years, and conditional failure risk does not fall to zero in long-term survivors [69,71]; a favourable 4-year PSA is associated with a low risk of recurrence, but this is a prognostic association and not evidence that surveillance can safely be reduced; it does not justify universal discharge, particularly in unfavourable intermediate-risk disease, and any reduction in surveillance intensity would require prospective validation. Identifying reliable early markers—nPSA12, early failure timing, and consecutive-rise dynamics—remains valuable for flagging high-risk patients for timely restaging and salvage treatments while distinguishing benign fluctuation from true recurrence.

#### 3.1.9. PSA Nadir Depth Across Treatment Modalities

Among the definitive radiotherapy modalities, LDR-BT has been reported to reach the lowest absolute PSA nadir, but this advantage in nadir depth does not translate uniformly into superior biochemical control. In the largest ADT-free comparison (3502 patients), median nadir was lowest after LDR-BT (0.01–0.2 ng/mL, reached at 51 months), intermediate after HDR-BT (0.1–0.2 ng/mL at 37 months), and highest after SBRT (0.2 ng/mL at 44 months), with LDR-BT reaching a nadir < 0.2 ng/mL in 72% of patients vs. 56% (HDR-BT) and 48% (SBRT); critically, however, biochemical recurrence-free survival was equivalent across all three ablative modalities, and there was no difference in the proportion reaching a nadir < 0.4 ng/mL at 4 years [17]. Because time to nadir differs by modality, cross-modality nadir comparisons at any single timepoint are confounded by the depth of continued late decay, as well as by differences in patient selection, risk group, ADT use, dose and follow-up.

This dissociation between nadir depth and biochemical control among ablative modalities is consistent across studies. LDR-BT and SBRT produced equivalent 5-year control (91.6% vs. 97.6%, *p* = 0.108), despite LDR-BT’s lower nadir [17]; HDR-BT ± EBRT and LDR-BT ± EBRT were equivalent after propensity weighting [74]; and in a pooled analysis of five prospective trials, HDR-BT was associated with a higher, not lower, risk of biochemical failure than was SBRT, despite HDR-BT’s deeper nadir [75]. Most strikingly, single-fraction 19 Gy HDR-BT was closed early for inferior control (4-year PSA control of 21% vs. 78% for LDR-BT), even though it produces frequent, large PSA excursions [76]. Nadir depth, therefore, tracks with outcome within a given modality but is not a valid surrogate for comparing biochemical control between ablative modalities.

The comparison against conventionally fractionated and moderately hypofractionated EBRT is different: here, the lower nadir achieved by LDR-BT does correspond to better biochemical control. LDR-BT was associated with a lower nadir than was EBRT (median of 0.10 vs. 0.40 ng/mL in one direct comparison, *p* < 0.0001) [65], accompanied by better biochemical outcomes in several comparative series—LDR-BT and HDR-BT were independently associated with lower biochemical failure than was 70.2 Gy EBRT [45]; propensity-matched ProCaRS (Prostate Cancer Results Study Group) analysis showed that brachytherapy improved biochemical failure-free survival across low- and intermediate-risk disease (HR up to 4.58) [77]; and LDR-BT was superior to image-guided IMRT (5-year biochemical failure free survival of 96.7% vs. 88.7%) [78]. In the randomized ASCENDE-RT (Androgen Suppression Combined with Elective Nodal and Dose Escalated Radiotherapy) trial, an LDR-BT boost halved the rate of biochemical failure relative to that of a dose-escalated EBRT boost (10-year freedom from progression of 85% vs. 67%), although without a significant difference in distant metastasis or overall survival [79]. Against proton-beam boost, LDR-BT again achieved a greater proportion of patients reaching a nadir ≤ 0.5 ng/mL (91% vs. 59%) with equivalent bRFS [41].

#### 3.1.10. Factors Affecting PSA Nadir Depth

Post-treatment PSA levels and nadir depth reflect the interplay of three factors: treatment intensity, tumour burden, and patient physiology. Treatment factors are dominant, because nadir depth is largely a function of residual PSA-secreting tissue and thus, of the delivered dose.

The specific dosimetric measures associated with PSA kinetics are minimum dose received by 90% of the prostate volume (D90), volume receiving 100% of the prescription dose (V100), and BED. A robust, plateauing dose–response links BED and dosimetric coverage to both nadir depth and biochemical control. In a 1377-patient series, 10-year freedom from PSA failure rose step-wise from 46% (BED < 100 Gy_2_ (biologically effective dose in gray, calculated using an α/β ratio of 2 Gy)) to ~90% and plateaued above 160–180 Gy_2_ [80], and post-treatment positive biopsy fell in parallel; larger analyses confirm BED ≥ 180 Gy_2_ as an independent predictor of biochemical control [57,67], with the caveat that requirements are risk-stratified—approximately ≥ 140–150 Gy_2_ suffices for low-risk disease, whereas high-risk disease may require ≥ 200 Gy_2_ [67]. Contemporary work quantifies the ceiling: BED ≥ 180–200 Gy_2_ is optimal, but ≥200 Gy_2_ increases Grade ≥ 3 toxicity without further oncologic gain, and in EBRT + BT combinations, no benefit is seen beyond ~ 220 Gy_2_ [45,81]. Dose escalation above the level needed to ablate even radioresistant benign tissue therefore provides no additional benefit and increases toxicity. Implant quality behaves similarly: a prostate D90 ≥ 90% of the prescribed dose (4-year bRFS 92.4% vs. 80.4%) and D90 > 140 Gy (10-year control 76% vs. 68%) predict superior control [82], and the American Society for Therapeutic Radiology and Oncology (ASTRO) endorses D90 ≥ 90% and V100 ≥ 85% as quality benchmarks [83]. Intraoperative real-time planning lowers nadir relative to preoperative planning [31,48]. Isolated null reports of no D90/V100 association (e.g., Franca et al.) likely reflect small, well-covered cohorts in which coverage exceeded the response threshold rather than the true absence of a dose effect [68]. SBRT and other high-BED regimens produce correspondingly low nadirs [17,42], whereas conventionally fractionated EBRT yields the highest [45]. These associations derive largely from cohorts in which adjustment for tumour characteristics was variable; implant-quality effects were generally independent of risk group, but few analyses simultaneously adjusted for testosterone/ADT status and time to nadir. A lower PSA nadir should not be read as direct evidence of superior dosimetry: nadir depth is jointly determined by delivered dose, residual tumour burden, testosterone status, and the duration of decline, so dosimetric superiority can be inferred only when these covariates are held constant.

Tumour-burden factors act in the opposite direction: higher iPSA, clinical T stage, Gleason score (GS), and risk group are each associated with higher post-treatment PSA and nadir values across modalities [18,19,28,32,35,39], reflecting greater malignant volume and PSA output.

Patient-physiology factors modulate interpretation. Older age correlates with lower nadir [65], plausibly through reduced benign epithelial vitality, and a longer time to nadir yields a lower final nadir [37]. Testosterone status is the most important interpretive caveat and warrants explicit documentation: subcastrate or incompletely recovered testosterone artificially depresses PSA, so a low nadir in a hypogonadal or recently ADT-treated patient overstates response. EBRT alone transiently lowers testosterone in up to 75% of men [84], and ADT more profoundly so, and nadir depth was prognostic only in men with normal testosterone at failure [64]. Whether deeper castration improves outcomes is genuinely unsettled— in one cohort, serum testosterone < 20 ng/dL was associated with lower nadir PSA and fewer recurrences [85], yet a randomized comparison found no difference between <20 and 20–49 ng/dL [86]. PSA bounce is a further confounder, transiently elevating intermediate-term PSA in bouncers, despite ultimately lower long-term nadir [18,28]. Failure to index PSA to testosterone may cause radiotherapy series to underestimate true BCR [64]. Longer follow-up attenuates all of these confounders.

### 3.2. PSA Bounce

#### 3.2.1. Definition and Proposed Mechanisms

PSA bounce—a transient rise in serum PSA above a prior post-radiation nadir with spontaneous return to or below that nadir without intervention—is characteristic of RT for PC, reported after LDR-BT, HDR-BT, CF-EBRT, H-EBRT, and SBRT. It is not a recognized feature after RP, and transient post-treatment rises after cryotherapy or high-intensity focused ultrasound are not well characterized. No universally accepted definition exists; historical criteria include any rise above nadir, absolute thresholds of ≥0.1, 0.2, 0.4, or 0.5 ng/mL, and relative increases of ≥15% or ≥35% from the prior value.

The most widely used criterion is a rise of ≥0.2 ng/mL above the current (pre-rise) nadir with subsequent spontaneous decline. Its preference is primarily empirical: across head-to-head comparisons of competing definitions, nadir + 0.2 ng/mL produces the fewest false-positive biochemical-failure calls (Zwahlen et al.) and was the only threshold among four for which the presence of a bounce significantly separated biochemical relapse-free survival (Caloglu et al., 5-year bRFS of 97.7% vs. 91%) [87,88]. This threshold also approximates the analytical and short-term biological variability of the PSA assay: within-subject variation is on the order of a 7–20% coefficient of variation, yielding reference-change values of roughly 15–50%, so a 0.2 ng/mL increment provides a practical margin above assay and physiologic noise at the low PSA values seen after BT [89,90]. The absolute 0.2 ng/mL cutoff should be understood as a pragmatic approximation rather than a metrologically derived constant, since biological variation is proportional rather than fixed.

Two measurement factors constrain interpretation of small rises at the low PSA values seen after LDR-BT. First is the above mentioned assay variability and inter-assay/inter-laboratory differences. At a nadir of 0.1–0.2 ng/mL, a 0.2 ng/mL increment lies only modestly above the combined analytical and physiologic noise, and serial values should ideally be obtained on the same assay platform and confirmed on repeat before being treated as a true rise. Second is testing frequency—more frequent PSA sampling inflates detected bounce incidence [88], while sparse sampling inflates apparent bounce duration [12]. Reported incidence, timing, and duration must therefore be interpreted in light of each cohort’s assay and testing schedule, and consecutive-rise definitions require standardized sampling intervals (see Section 3.2.8).

The biological mechanism remains unproven and is likely multifactorial. The evidence supporting each of the hypotheses below is correlative, and none establishes causation. Non-radiation contributors—physiologic variation, assay variability, recent ejaculation, urinary-tract instrumentation, proctitis, cycling, and testosterone recovery after ADT—must be excluded, and several series specifically exclude concurrent urinary infection or prostatitis [88,91]. Three principal radiation-related hypotheses have distinct supporting evidence: 1—a delayed inflammatory/prostatitis reaction, supported by 3D MR-spectroscopic imaging showing diffuse prostatic metabolic activity during the bounce that is unrelated to residual tumour [92]; 2—a slow sublethal-to-lethal transition in irradiated benign and malignant cells producing a clustered release of intracellular PSA [93]; and 3—a delayed cell-mediated immune response, supported by higher intratumoural CD3/CD8 T-cell density in bounce-positive patients, a fitted tumour–immune dynamics model, and elevated peripheral activated T cells after brachytherapy [94]. The immune hypothesis is of particular interest because it is compatible with the association between bounce and improved biochemical control and with the lower bounce incidence in older patients, who may have reduced immune competence; however, these are associations, the proposed mechanisms are not mutually exclusive, and none has been established as the mechanism of bounce. Beyond radiotherapy, high-intensity focused ultrasound has been reported to modulate the prostate tumour microenvironment, inducing immunogenic cell death [95]. Similar immune-related changes after thermal and radiation ablation raise the possibility of a shared cell-mediated component in post-ablation tissue responses, but this remains speculative.

Paralleling these thermal-ablation dynamics with radiation-induced immune responses highlights a shared cell-mediated mechanism underlying post-ablation tissue responses and transient biomarker fluctuations in prostate cancer.

#### 3.2.2. Bounce Incidence

Reported bounce incidence depends heavily on the threshold definition, follow-up duration, PSA sampling frequency, and whether rates are actuarial or crude. The incidence falls monotonically as the threshold rises: in a single large series, it was 41.3%, 28.4%, 17.4%, and 6.8% at nadir + 0.2, +0.5, +1.0, and +2.0 ng/mL, respectively [96], and 30.1%, 19.6%, 12.8%, and 9.5% at nadir + 0.2, +0.4, +0.6, and +0.8 ng/mL in an 820-patient LDR-BT cohort [88]. At the lowest (nadir + 0.1 ng/mL) definition, crude incidence reaches 48.5–55% [24,97,98] vs. a 5-year actuarial rate of ~ 31% [39]. When the standard nadir + 0.2 ng/mL bounce definition is utilised, reported bounce rates across an LDR-BT series range from 13–54.2%, with a median of 33.2% [10,11,12,19,24,25,54,87,88,91,97,99,100,101,102,103,104,105,106,107,108,109,110,111], consistent with the 27.4–50% range reported in the meta-analysis by Bernstein et al. [7]. A comprehensive study-level summary detailing sample sizes, patient age distributions, radioisotopes, treatment modalities, definitions, follow-up, testing schedules, and reported bounce rates across individual series is presented in Supplementary Table S2. The observed variation across studies is driven by specific clinical and methodological determinants:

- Patient Age: Younger patient age (<60 years) is the dominant clinical driver of higher bounce rates (e.g., 51.8–54.2% in younger cohorts vs. 24.3–27.7% in older cohorts) [103,104,106].
- Radioisotope (Dose Rate): Slower dose-rate permanent radioisotopes (^125^I, half-life ~ 60 days) exhibit higher bounce rates than those of faster dose-rate isotopes (^103^Pd, half-life ~ 17 days), at 45.7% vs. 14.0% for hormone-naive patients in a randomised trail [112].
- Treatment Modality: LDR monotherapy yields higher bounce rates than those of combination regimens (LDR + EBRT)—Tanaka et al. reported 28% vs. 18% [108].
- PSA Sampling Frequency: Schedules with high sampling density (e.g., quarterly vs. annual testing) detect more transient spikes that would otherwise be missed using more distant intervals [88].

At nadir + 0.4 ng/mL, bounce incidence is reported in the range of 18.4–34% [24,26,87,88,97,98,111].

Bounces exceeding the Phoenix threshold (nadir + 2 ng/mL) are clinically important because they mimic failure. Their incidence is 2.5–18%, representing 7.5–24% of all nadir + 0.2 ng/mL bounces [12,24,87,100,104,107,109,113,114,115,116]. Critically, a substantial fraction of Phoenix-triggering rises are benign: with adequate follow-up, 44% of Phoenix ‘failures’ in a 1006-patient cohort proved to be bounces [116], and across series, 20–50% of nadir + 2 ng/mL crossings resolve spontaneously [24,87,97,104,116].

Sequential bounces are less common and diminish sharply with each event. A second bounce occurs in ~4–5% of patients (median ~ 2.5 years post-treatment; in limited data, with a median magnitude of 0.18 ng/mL and duration ~ 6 months), and a third in ~0.5% [24,106,108].

#### 3.2.3. Predictors of PSA Bounce

The wide heterogeneity in reported bounce incidence after LDR-BT is multifactorial, reflecting a combination of patient, tumour, treatment, and methodological influences.

Younger age is the most consistent and strongest independent predictor of PSA bounce across all RT modalities, a finding supported by a meta-analysis of 26,258 patients [9]. Its effect can be large in illustrative cohorts, with an incidence of ~20% in men < 65 years vs. ~4% in men > 65 years [19]. However, in cross-modality comparisons, patient age assumes a secondary role, as choice of treatment modality becomes the primary driver of observed variation. In the same meta-analysis, treatment modality explained the largest single share of heterogeneity in bounce incidence (~30%), ahead of age (~20%) and risk classification (~12%) [9]. Age is also partially confounded by correlated variables, i.e., prostate and transition-zone volume, baseline PSA, and risk category. Nevertheless, in a major multivariate modelling series, when age was pitted directly against prostate volume, D90, V100, volume receiving 150% of the prescription dose (V150), iPSA, GS, and ADT status, all gland and treatment variables dropped out, leaving age as the only surviving independent factor [12,24,91,103,117]. While younger age remains the most robust independent predictor across multivariate analyses, its effect reflects a combination of host physiological factors—such as immune responsiveness and androgen status—interacting alongside gland volume and dose distribution rather than operating as an isolated demographic variable.

Younger age also shapes the character of the bounce, as younger patients reach lower pre-bounce nadirs, experience more sequential bounces, and develop higher-magnitude spikes, including those crossing the Phoenix threshold; patients with high-amplitude, Phoenix-triggering bounces are also significantly younger than those with true BCR [107,116,118]. Better-preserved sexual function is an independent correlate, consistent with the greater physiologic integrity of younger men [111]. The same age effect is observed after EBRT, underscoring that it is patient- rather than modality-driven [119].

In contrast to age, the relationship between pre-treatment tumour burden and prostate volume with post-treatment PSA bounce incidence is weaker and inconsistent. Across published series, indicators of lower tumour burden generally correlate with higher bounce rates via univariate analysis, but their independent predictive power is often lost when adjusted for patient age [6,107]. The indicators evaluated across series include clinical T stage, Gleason grade group, initial PSA, composite risk category, Cancer of the Prostate Risk Assessment (CAPRA) score, percentage of positive biopsy cores, and prostate/transition-zone volume.

Lower histopathological grade (Gleason score 6/Grade Group 1) has been associated with PSA bounce via univariate analysis [19,104,115]; however, across multivariate logistic models restricted to pure LDR-BT monotherapy, GS routinely loses statistical significance and drops out of the final regression models [12,24,98,107]. Lower baseline iPSA levels exhibit unadjusted inverse associations with bounce frequency in several LDR-BT series [104,108]. Nevertheless, iPSA consistently fails to maintain independent prognostic value in multivariate analyses once adjusted for concurrent clinical factors [12,24,98,107]. Clinical stage (cT1c vs. cT2) showed a univariate trend in one series [6], but fails to predict PSA bounce independently in multivariate regression models [24,98,103,107]. In a dedicated multivariate analysis of younger patients, Charret et al. reported a lower positive-to-total biopsy ratio as an independent predictor of PSA bounce (HR 0.98, 95% CI: 0.97–0.99; *p* = 0.037) [120]. While isolated series identified prostate volume [87,107] or transition zone index [121] as independent predictors, in multi-variable analyses, prostate volume routinely loses statistical significance and drops out as an independent predictor when adjusting for concurrent clinical parameters [12,24,103].

Unlike pure LDR-BT monotherapy, in a mixed treatment modality cohort of LDR- and HDR-BT, GS was predictive of bounce occurrence via multivariate logistic regression (OR 2.21, 95% CI: 1.5–3.3; *p* < 0.0001) [105]. The same association was reported in an EBRT cohort (*p* = 0.0003) [119]. In multi-modal comparisons evaluating standard EBRT, hypofractionated EBRT, and brachytherapy, multivariate modelling demonstrates that a lower CAPRA score (CAPRA 1 vs. 3: OR 0.34, 95% CI: 0.15–0.73; *p* = 0.007), and a lower percentage of positive biopsy cores (*p* = 0.039) remain independent multivariate predictors of PSA bounce [122]. The 2020 meta-analysis likewise identified lower risk group as an independent bounce predictor [9]. The predictive role of tumour-burden-related parameters—specifically GS, iPSA, clinical stage, and risk category—remains inconclusive.

No consistent dosimetric predictor of bounce has emerged. The measures tested—D90, V100, V150, V200—show no reproducible association with bounce incidence in most series [24,104], and the isolated positive signals conflict with one another: higher urethral dose (%UD90) [108] and D90 > 200 Gy [97] have each been linked to more bounces, whereas, in another cohort, lower D90/V150 in regards to the dominant intraprostatic lesion has been linked to more bounces [25]. While each of these isolated signals was identified in single-institution multivariable models, none has been consistently replicated in independent external validation cohorts. The choice of isotope may also contribute; in a single hormone-naive subgroup, bounce incidence was higher with ^125^I than with ^103^Pd (45.7% vs. 14%), suggesting that slower dose-rate delivery favours the phenomenon [112].

Where individual dosimetric parameters fail as predictors, the treatment modality itself is the single strongest determinant of bounce, accounting for approximately 30% of the between-study heterogeneity [9]. Reported incidence ranks carbon ion radiotherapy (56%) > HDR-BT monotherapy (36%) ≈ LDR-BT monotherapy (34%) > SBRT (28%) ≈ EBRT + BT (28%) > EBRT (22%) [9], with a corroborating ordering of LDR-BT (42%) > HDR-BT (23%) > EBRT (20%) reported elsewhere. Consistent with this, bounce is more common after BT than after EBRT [9] and more common after ^125^I LDR-BT than after EBRT ± HDR-BT or LDR-BT combined with EBRT [11,45]. The highest rates occur with single-fraction 19 Gy HDR-BT, despite its relatively low BED [117]. The observation that the two kinetic extremes—the protracted delivery of ^125^I LDR-BT and the ultra-rapid delivery of single-fraction HDR-BT—both produce high bounce rates implies that more than one radiobiological pathway can generate the phenomenon. However, attributing these observed discrepancies directly or solely to treatment delivery technique requires cautious interpretation. They should be interpreted as aggregate signals rather than direct causal effects of modality per se. Reported differences between LDR-BT, hypofractionated EBRT, and conventionally fractionated EBRT are confounded by differences in dose per fraction and total BED, patient selection (younger, lower-risk patients are preferentially offered brachytherapy), PSA sampling frequency, follow-up duration, and the bounce definition applied. LDR-BT monotherapy cohorts are systematically enriched with younger, hormone-naïve patients. Because younger age independently increases bounce probability [9], unadjusted comparisons between LDR-BT cohorts and older, EBRT cohorts inherently overestimate the intrinsic bounce propensity of LDR-BT.

ADT appears to have little independent effect on bounce frequency. Most series, as well as the multimodality meta-analysis, find no association between ADT and bounce incidence [9,103,110]. The effect of ADT on PSA interpretation operates at three distinct points and should not be conflated with a true bounce. During treatment, ADT suppresses androgen-dependent PSA production, driving an early, partly pharmacologic nadir, so early thresholds derived from ADT-free cohorts cannot be transferred to ADT-treated patients, or vice versa [73]. After cessation, testosterone recovery (non-castrate levels within 6–15 months; full eugonadal recovery, often over years) drives a testosterone-dependent PSA rise that can mimic bounce or early recurrence and can confound kinetics for up to ~3 years [104,116]. Documenting ADT exposure, cessation date, and a concurrent serum testosterone is therefore essential to interpreting any post-treatment PSA rise.

Finally, much of the apparent variability across studies is methodological rather than biological, and comparisons must therefore be standardized. Fluctuations within the first three months—driven by needle trauma, prostatic oedema, catheterization, or localized infection—are not radiation-induced bounces and should be excluded [24,87]. More frequent PSA sampling inflates the detected incidence [88], whereas defining a bounce only after the absolute nadir has been reached underestimates it by 50% or more [96]; accordingly, a bounce should be referenced to the current (pre-rise, nadir-to-date) PSA rather than to the absolute nadir [20]. Reported racial differences (42% in African American vs. 29% in Caucasian men) may reflect host or genomic differences rather than proven genetic causation [123]. Post-treatment PSA kinetic indices, including PSA doubling time and PSA velocity, do not predict bounce [24].

#### 3.2.4. Time to Bounce

The approximately 1.5-year median time to bounce reported here is a descriptive summary of per-study medians, not a pooled estimate; across the LDR-BT literature, individual-series medians fall between 12 and 24 months (with most at 14–20 months) [10,12,19,24,54,61,87,88,91,97,99,106,107,108,109,111,115], concordant with the 17.4–19.5-month range of the 2013 meta-analysis of 3011 patients treated predominantly with ^125^I LDR-BT [7]. The 1.5-year value was summarized narratively; no pooling or meta-regression was performed in the present review.

The choice of nadir-plus-threshold definition has little effect on timing—the difference in median onset between the nadir + 0.2 ng/mL and nadir + 0.4 ng/mL definitions is not significant [24,87,88,111]—and even high-magnitude spikes that reach the Phoenix threshold do so on a similar schedule, with a median time to cross the nadir + 2 ng/mL threshold of 17.5–20.6 months [107,114,116]. Bounces cluster early: more than 80% occur within the first 2 years [111], more than 90% within 3 years [24,88,107,109], and more than 95% within 5 years [10,88,97].

True biochemical recurrence consistently manifests later than PSA bounce, regardless of modality, though the two windows overlap substantially [12,24,61,87,88,110,123,124,125]. The reported median time for patients with BCR to cross the nadir + 2 ng/mL threshold after LDR-BT is 27.8 to 55.3 months [12,24,54,61,87,88,104,107,114,116]. Both a delayed onset and a prolonged duration of PSA elevation correlate with higher BCR risk: Lo et al. reported that the incidence of BCR among elevations present during years 2–3, 3–4, and the sustained window of years 2–4 rose to 52%, 79%, and 100%, respectively [35]. A complementary kinetic signal was described in the work of Thompson et al., in which bouncers reached their initial pre-bounce nadir faster than patients with BCR reached their pre-failure nadir (median of 6 vs. 11.5 months) [116]—plausibly because persistent low-level secretion by recurrent malignant cells protracts the pre-failure decline. Timing of onset, therefore, remains the single most robust feature distinguishing a benign bounce from true recurrence, and late-occurring or sustained elevations should be regarded as recurrence until proven otherwise.

Most clinical variables do not reliably predict when a bounce will occur. Race influences neither time to bounce nor time to BCR [123]. Single-institution analyses have linked shorter latency to lower CAPRA score [122], younger age [118], and ADT use [126], while a large systematic meta-analysis, without an LDR-BT-specific subgroup, did not find such associations and identified treatment modality as the only predictor of bounce timing, with SBRT and HDR-BT showing shorter latency than that of LDR-BT and CF-EBRT [9]. Consistent with this, Pickles et al. found a shorter time to bounce after LDR-BT than after EBRT [126]; that HDR-BT precedes LDR-BT [105]; and that HDR-BT, SBRT, and SBRT + EBRT report comparable timing [9,127,128]. Dose rate also contributes: in hormone-naive patients, ^103^Pd did not shorten latency relative to ^125^I, but with ADT, only ^103^Pd-treated patients showed accelerated onset, whereas ^125^I timing was unaffected by ADT status [112].

To date, treatment modality is the only determinant of timing identified in the available meta-analysis [9], and the pattern may plausibly be explained radiobiologically, although this explanation is speculative: ablative doses delivered in one to several fractions (HDR-BT, SBRT) produce the shortest latency, the protracted delivery of ^125^I LDR-BT reaches ablative dose more gradually and yields intermediate onset, and CF-EBRT—despite delivering its total dose over a shorter calendar period—produces the latest onset, through delayed mitotic cell death, at a lower dose per fraction and lower BED. The roles of age, tumour burden, and ADT in bounce timing remain unresolved and warrant dedicated, LDR-BT-specific investigation.

#### 3.2.5. Bounce Duration

Descriptively summarized across published cohorts, post-LDR-BT PSA bounces exhibit a median duration of approximately one year, with reported study medians clustering tightly around this interval and ranging from 6.24 to 21 months [10,12,24,87,101,106,108,109,113]; the 1-year median duration was not derived by quantitative pooling. The distribution is right-skewed: more than 75% of bounces resolve within 2 years, while only about 10% to 18.3% persist beyond 2 years [24,109]. The trajectory is broadly symmetrical, with the ascending-limb and descending-limb shoulders of the bounce occupying roughly equal time [24]. Bounce duration is prolonged by amplitude—a higher-magnitude bounces require longer to resolve [96]. This relationship is bounded, however, rather than proportional: even large-magnitude spikes last only modestly longer than smaller ones. Kubo et al. illustrated this ceiling, reporting a median duration of 4.5 months (maximum of 1 year) for the entire interval from crossing the nadir + 2 ng/mL threshold to declining back below it [107]. Thus even Phoenix-crossing bounces are typically self-limited and short-lived. Beyond magnitude, longer bounces are seen with ADT [96], although neoadjuvant ADT specifically showed no effect on duration, in some series [108]. Younger age is also associated with longer-lasting bounces [118]. A distinct interaction exists between ADT and isotope dose rate: Bostancic et al. found an identical mean bounce duration between hormone-naive patients treated with ^125^I and ^103^Pd, but in the ADT subgroup, ^125^I produced a mean duration roughly 4 months longer than that for ^103^Pd, implicating the slower ^125^I dose rate [112]. Dosimetric parameters and BED otherwise show no significant influence on duration. Duration is comparable across the high-dose-per-fraction and low-dose-rate modalities—HDR-BT and SBRT yield a bounce duration similar to that of LDR-BT [124,129]—whereas EBRT produces shorter-lasting bounces [89]. As with incidence, sparse sampling inflates the apparent bounce period, since infrequent measurements can artificially extend the interval over which a bounce is deemed active [12]. Reported duration should therefore be interpreted in light of each cohort’s PSA testing schedule.

#### 3.2.6. Bounce Magnitude

The median height of a PSA bounce ranges from 0.29 to 1.4 ng/mL across series, most commonly clustering between 0.5 and 0.9 ng/mL [10,12,19,24,61,87,91,99,101,103,104,105,106,108,111,115,122]. Reported magnitude is definition-dependent: applying a higher threshold selects for larger events and inflates the median amplitude [87]. Most bounces remain under 1 ng/mL, but a minority markedly exceed the Phoenix threshold and can, on rare occasions, surpass the iPSA.

In a multimodality meta-analysis, younger age was the only statistically significant predictor of higher bounce magnitude [9]. This pattern is compatible with, but does not demonstrate, the immune-mediated framework proposed for the phenomenon, under which larger rises in younger men would be expected to accompany a stronger radiation-enhanced inflammatory response.

Reported bounce amplitude is higher after hypofractionated schedules and brachytherapy than after CF-EBRT. Waters et al. reported higher magnitudes for LDR-BT and H-EBRT than for CF-EBRT (0.64, 0.60, and 0.38 ng/mL, respectively) [122], and the pooled meta-analysis by Darwis et al. found the highest mean amplitude for LDR-BT (1.7 ng/mL), followed by HDR-BT (1.4 ng/mL), SBRT (1.0 ng/mL), and EBRT (0.8 ng/mL) [9]. Within HDR-BT, single-fraction 19 Gy produces larger bounces than those for multifraction regimens [117]. Taken together, these data suggest that bounce amplitude is higher after LDR-BT and hypofractionated schedules, most notably single-fraction 19 Gy HDR-BT, than after CF-EBRT. These are between-study associations: the comparisons are not adjusted for age, sampling frequency or follow-up (see Section 3.2.3). Table 1 summarizes the impact of clinical-, patient-, and treatment-related predictors on various parameters of post-treatment PSA dynamics.

Endpoints: time to nadir, interval from treatment to the lowest post-treatment PSA (nPSA); absolute nadir, the nPSA value (ng/mL); bounce incidence, proportion of patients with a transient PSA rise ≥ 0.2 ng/mL above the current nadir (nadir + 0.2 ng/mL) that returns spontaneously to or below that nadir; time to bounce onset, interval from treatment to the PSA rise meeting the bounce definition; bounce duration, interval from onset until PSA returns to or below the pre-bounce nadir; bounce magnitude, peak PSA minus the pre-bounce nadir (ng/mL).

Abbreviations: ADT, androgen deprivation therapy; BED, biologically effective dose; CF-EBRT, conventionally fractionated external beam radiotherapy; D90, minimum dose covering 90% of the prostate volume; GS, Gleason score; H-EBRT, hypofractionated external beam radiotherapy; HDR-BT, high-dose-rate brachytherapy; iPSA, initial (pretreatment) PSA; LDR-BT, low-dose-rate brachytherapy; N/E, not evaluated; nPSA, PSA nadir; NS, not significant; PSA, prostate-specific antigen; SBRT, stereotactic body radiation therapy; T stage, clinical tumour stage; V100, percentage of the prostate volume receiving 100% of the prescribed dose; 125I, iodine-125; 103Pd, palladium-103.

#### 3.2.7. PSA Velocity: PSA Bounce vs. Biochemical Recurrence

The kinetic parameters of a rising PSA—PSA velocity and PSA doubling time (PSADT)—have been evaluated extensively as tools to separate a benign bounce from true BCR, with predominantly disappointing results. In most series, PSADT does not differ significantly between bouncing patients and those meeting the Phoenix or ASTRO definitions of BCR [110,115]. Mitchell et al. reported a higher median PSA velocity during Phoenix failures than during bounces (0.28 vs. 0.08 ng/mL/month), but with no usable cutoff because the distributions overlapped [115]. Thompson et al. found the opposite direction on doubling time, with BCR patients showing a longer median PSADT than Phoenix-crossing bounces (6.9 vs. 5.1 months), alongside lower pre-rise nadirs [116]. These conflicting directions across cohorts are themselves the central finding: no consistent velocity or doubling-time threshold reliably discriminates the two events.

A coherent kinetic rationale underlies the poor discrimination. Bounces typically show a rapid initial rise, which tends to produce a shorter PSADT and a higher instantaneous velocity than those for BCR; in principle, this should distinguish them. In practice, the difference between benign bounce and true failure is too small relative to the biological and assay-related scatter, so the distributions overlap heavily, and individual studies report contradictory results. This overlap undermines the clinical utility of early velocity metrics for real-time decision making.

The recurrent observation of a lower pre-rise nadir in patients who ultimately fail is consistent with post-radiation PSA dynamics rather than indicative of a true kinetic discriminator. Bouncers experience their PSA rise earlier, leaving less time for the PSA to decay before the rise, so their pre-rise nadir is comparatively higher. Patients destined for BCR undergo a more protracted initial decline—reflecting persistent low-level secretion by residual malignant cells—before regrowth reverses the trajectory and therefore, reach a lower nadir before the eventual rise.

#### 3.2.8. Prognostic Significance of PSA Bounce

The preponderance of the evidence establishes PSA bounce as a favourable prognostic sign following definitive radiotherapy. Numerous LDR-BT series, together with a systematic review and meta-analysis of 8881 patients treated with definitive RT, have linked the occurrence of a bounce to a reduced rate of BCR, with the meta-analysis reporting this prognostic association for both LDR-BT and EBRT monotherapy [7,8,24,54,60,61,63,87,88,91,103,104,106,109,115]. Beyond BCR, some series associate bounce with a decreased risk of clinical progression and with improved 10-year disease-specific and overall survival [103,109].

The protective association is strongest for the standard nadir + 0.2 ng/mL definition and attenuates as the bounce threshold rises. Bounces defined by higher thresholds lose statistical significance in relation to BCR [87,88], most plausibly because raising the cutoff reduces bounce incidence and therefore, statistical power. A qualitatively different signal emerges at the highest amplitudes: McGrath et al. found bounces above nadir + 1 ng/mL to carry a non-significant increase in clinical failure risk, whereas bounces < 1 ng/mL showed no such trend, prompting the hypothesis that some large spikes reflect occult disease or greater residual tumour volume rather than a purely benign bounce [96]. This suggests that the favourable prognostic meaning of a bounce should not be extrapolated uncritically to very high-magnitude events.

Not all series confirm the association [21]. Charret et al., studying men younger than 60 years, found no relationship between bounce and BCR but did observe an association with grade 2–3 urinary toxicity [120]. Two non-exclusive explanations are plausible: the low failure rate in young, favourable-risk cohorts may leave insufficient events to demonstrate a protective effect, or the greater biological aggressiveness of early-onset disease may offset it. These null results are therefore best interpreted as limited power or population-specific effects rather than refutation of the overall trend.

The protective benefit of bounce has been reported as more pronounced in African American than in Caucasian patients [123]. One hypothesis, which remains speculative, is that bounces in African American patients could be accompanied by greater intratumoural immune infiltration and radiosensitivity, whereas bounces in Caucasian patients may be associated with predominantly benign prostatic tissue inflammation. This mechanistic distinction is not established and should be presented as a direction for investigation rather than as a conclusion.

Because bouncing patients undergo more frequent PSA testing during active spikes [88], consecutive-rise BCR definitions are vulnerable to detection bias. Testing intervals should be standardized and ideally, specified within the definition itself. A risk-adapted schedule keyed to post-treatment PSA velocity could further reduce this bias.

### 3.3. Definitions of BCR

#### 3.3.1. Threshold Definitions

Because LDR-BT delivers a high BED and drives PSA to near-undetectable levels comparable to those after RP, the choice between the RP (‘surgical’) definition and the Phoenix definition is more consequential than it is after EBRT. The American Urological Association surgical definition—PSA ≥ 0.2 ng/mL confirmed on a second measurement—is attractive after LDR-BT because it permits direct, lead-time-unbiased comparison of biochemical control rates against RP [56,83,130]. Its inappropriateness for CF-EBRT, where residual benign tissue sustains a much higher nadir, is illustrated by the ASCENDE-RT analysis: applying the surgical rather than the Phoenix threshold roughly doubled 7-year biochemical failure in a 78 Gy CF-EBRT cohort, while making no difference in the LDR-BT boost arm [131].

Head-to-head data confirm that the two definitions perform comparably after LDR-BT but with distinct trade-offs. In 427 patients treated to 160 Gy, specificity for freedom from failure at 4 years was 100% for both definitions, and accuracy was similar (Phoenix, 94.8%; surgical, 96.3%); however, the negative predictive value was far lower for the surgical definition (7.7% vs. 29%), meaning that failing to reach a PSA ≤ 0.2 ng/mL is a weak indicator of true failure and should not by itself prompt salvage [132]. Conversely, the Phoenix nadir + 2 threshold can underestimate failure by missing a small group with a slowly rising PSA that remains below nadir + 2: in 2339 patients, nPSA and 60-month PSA were the strongest predictors of ultimate failure (odds ratios ~ 20), identifying failures that a strict nadir + 2 rule would miss [133]. These reciprocal limitations—the surgical definition over-calling failure and Phoenix under-calling it—comprise the central rationale for incorporating absolute low-PSA thresholds into post-LDR-BT surveillance.

The prognostic value of a low absolute PSA at adequate follow-up is robust and graded. In the 14,220-patient pooled analysis, a 4-year PSA ≤ 0.2 ng/mL predicted a 10- and 15-year freedom-from-recurrence of 98.7% and 96.1%, with step-wise worse outcomes across higher strata; the authors proposed a 4-year PSA ≤ 0.2 ng/mL as a biochemical definition of cure, a threshold endorsed in the American College of Radiology–American Brachytherapy Society–American Radium Society practice parameter precisely because it facilitates comparison with RP [66,83]. External validation confirmed a 10-year DFS of 97.5% for ≤0.2 vs. 41.8% for >1 ng/mL, but with important risk-group dependence—the 10-year DFS was 93.1% for low-risk and 92.1% for favourable-intermediate risk, yet only 75.9% for unfavourable-intermediate risk—so the prognostic value of this threshold, and any case for individualized de-escalation, is strongest in low- and favourable-intermediate-risk disease and has not been prospectively validated [69]. Lower thresholds carry even stronger specificity: a 5-year PSA < 0.1 ng/mL was associated with no BCR or clinical failure events (specificity of 100%; positive predictive value of 100%) and proposed by the authors as a possible criterion for reduced surveillance intensity [62].

These data support the risk- and time-stratified relaxation of monitoring. In 1569 patients, no low-risk patient with a year-3 PSA ≤ 0.2 ng/mL and no intermediate-risk patient with a year-5 PSA ≤ 0.2 ng/mL developed BCR over a median 8-year follow-up, suggesting, in these retrospective cohorts, that long-term PSA testing might be reduced once these thresholds are met; this has not been validated prospectively [16]. Two caveats govern all early-threshold rules: testosterone and ADT status. In a population-based cohort of 5299 men, a detectable PSA at 6–12 months predicted prostate-cancer mortality only among RT + ADT patients and not among those treated without ADT because ADT compresses the early decline [73]; thresholds derived from ADT-free brachytherapy cohorts therefore cannot be transferred to ADT-treated patients. Accordingly, PSA values of <0.1, 0.2, 0.4, and 0.5 ng/mL all function as surrogates for minimal residual benign and malignant tissue and are well-validated prognostic markers, provided follow-up is long enough to let PSA bounce resolve and radiobiological effect mature, and that provided testosterone status is documented.

The thresholds above describe the association between a PSA level at a given time and the subsequent outcome in retrospective cohorts. Using them to change follow-up (fewer tests; discharge) is a separate question that requires evidence on clinical endpoints and harms, including disease-specific survival and salvage efficacy. No threshold reported here has been validated prospectively for this purpose.

#### 3.3.2. Threshold Definitions vs. the Phoenix Definition

The surgical and Phoenix definitions applied to the same LDR-BT cohort yield systematically different recurrence rates because a minority of patients never reach PSA ≤ 0.2 ng/mL, even at adequate follow-up. Tsumura et al. showed that applying Phoenix definitions to an LDR-BT cohort produced a higher 8-year bRFS than RP, whereas applying the surgical definition to both erased the difference [134]. In 2634 patients, the Phoenix BCR (11.1%) was lower than the surgical BCR (17.5%), a gap significant at 5 and 10 but not 15 years, as patients with slowly rising levels eventually satisfy both definitions [135]. These divergences reflect reciprocal, opposite errors rather than a single bias: the surgical definition over-calls failure (its negative predictive value for freedom from failure was only 7.7% in the head-to-head comparison discussed above), while the Phoenix nadir + 2 threshold under-calls it, missing a small group with PSA rising slowly below nadir + 2 (nadir PSA and 60-month PSA being the strongest failure predictors; odds ratios ~ 20) [133].

The superiority of nadir-referenced over consecutive-rise definitions is well established. In pooled analyses of 4839 EBRT patients, nadir + 2 (dated at call) and current-nadir + 3 outperformed the ASTRO three-rises definition and were free of its backdating bias, and simulation work confirmed that nadir + 2 is more specific and less sensitive to sampling frequency and follow-up duration than are consecutive-rise rules [136,137,138]. Notably, the same analyses found fixed low thresholds (PSA > 0.2 or > 0.5 ng/mL) to be inferior to nadir-referenced definitions for predicting clinical failure [136], which is an important distinction from the prior section: a low absolute PSA is a strong prognostic marker of favourable outcome but a comparatively poor trigger for defining failure, for which a nadir-referenced rise performs better. All definitions also predict clinical failure more accurately after BT or neoadjuvant ADT than after EBRT alone because the lower post-BT nadir produces less background PSA ‘noise’ [139]—direct support for treating post-LDR-BT PSA as a distinct entity.

Each definition class carries a follow-up-dependent trade-off. The surgical threshold is vulnerable to early PSA bounces, which are most prevalent in the first years; nadir-plus-threshold definitions suppress this false-positive noise but lose sensitivity late. Kuban et al. (2693 LDR-BT patients) therefore proposed Phoenix for short follow-up and a nadir < 0.5 ng/mL for follow-up beyond ~ 6 years [10]. A contemporary refinement adds kinetic criteria to Phoenix: in 2038 SBRT patients, nadir + 2 had ~30% false-positivity that fell to 6.4% when combined with a relative-PSA-decline or time-to-nadir + 2 ≥ 18 months criterion—a direct precedent for the time-adapted framework proposed here [140].

The surgical threshold retains prognostic value across the other ablative, high-BED modalities. After HDR-BT boost, failure to reach an nPSA < 0.4 ng/mL predicted markedly worse control (5-year bRFS of 100% vs. 72%) [57]; in HDR-BT monotherapy, nadir ≤ 0.2 ng/mL predicted a 10-year bRFS of 96.9% vs. 40% [141]; and long-term series report very low recurrence risk once PSA is ≤0.2 ng/mL at 7–8 years after HDR-BT, which some authors have taken to support discontinuing surveillance [142]. This convergence across LDR-BT and HDR-BT reinforces that absolute low-PSA thresholds are consistently associated with a favourable outcome wherever an ablative dose is delivered.

#### 3.3.3. Alternative Definitions

The nadir + 2.0 ng/mL benchmark was selected from a cross-evaluation of candidate thresholds (nadir + 1.0, +2.0, +3.0) as the compromise that maximized specificity while accepting some loss of sensitivity: lower ceilings increase early, bounce-driven false positives, whereas higher ceilings delay recurrence detection [10,65,67]. Subsequent proposals have moved from this compromise in both directions—toward greater sensitivity with intermediate sub-integer thresholds, and toward greater specificity with high-ceiling, kinetics-based definitions.

The sensitivity-favouring direction is exemplified by a nadir + 0.4 ng/mL threshold assessed at year 3. In a single institutional cohort, all failures satisfied both nadir + 0.4 ng/mL and Phoenix, no nadir + 0.4 recurrence later regressed, and progression from nadir + 0.4 ng/mL to nadir + 2 was rapid (median 17 months; 70.6% within 2 years, faster with a higher Gleason score) [28]. However, univariate and multivariate models produced nearly identical hazard ratios and covariates for the two definitions, so nadir + 0.4 offers earlier calling—not superior prognostic discrimination. Whether that lead time improves salvage efficacy or disease-specific survival is unproven, and lowering the ceiling reintroduces the bounce-driven false positives that Phoenix was designed to suppress: benign bounces reach the nadir + 0.4 threshold in 18–34% of patients (see bounce-incidence section). The nadir + 0.4 ng/mL threshold should therefore be regarded as hypothesis-generating and requires prospective validation against clinical endpoints before adoption.

The specificity-favouring direction is exemplified by the Japanese Prostate Cancer Outcome Study (J-POPS) definition, developed within the nationwide Japanese prospective registry of permanent ^125^I seed implantation: PSA > 1.0 ng/mL rising across three consecutive measurements. It substantially reduces spurious failure calls—in 2316 patients, 91.1% of Phoenix ‘failures’ subsequently fell back below nadir + 2 ng/mL vs. only 22.2% of J-POPS failures [143]—and behaves as expected for a high-ceiling definition, yielding a higher bRFS than the that of surgical definition (5-year bRFS of 96.9% vs. 85.0% in a direct comparison) [144]. The reciprocal cost is that requiring a higher, sustained, rising PSA necessarily detects true recurrence later than does Phoenix, delaying salvage in genuine failures; its validation to date is against biochemical rather than clinical endpoints (metastasis, disease-specific survival), and its derivation using a ^125^I monotherapy population leaves generalizability to HDR-BT, EBRT, and combination therapy untested.

These opposite adjustments illustrate the ceiling-tuning dilemma: any single fixed threshold trades false positives against lead time. Kinetic augmentation offers a more robust alternative by conditioning the failure call on the shape of the PSA curve rather than using a single magnitude. In 2038 patients, adding a relative-PSA-decline criterion or a time-to-(nadir + 2 ng/mL) ≥ 18-month criterion to the Phoenix definition reduced its ~ 30% false-positive rate to 6.4% without sacrificing sensitivity [140]. This kinetics-conditioned approach—distinguishing the rapid, self-resolving trajectory of a bounce from the sustained rise of true recurrence—is the conceptual foundation of the time-adapted framework proposed below.

#### 3.3.4. Differentiating Local Failure from Metastatic Disease

No PSA metric reliably separates local persistence from distant progression; definitive determination requires imaging. PSADT remains the key kinetic discriminator and timing trigger: a short PSADT (<12, <6, and especially <3 months) predicts distant metastasis and cancer-specific mortality, including in the post-radiation Phoenix-failure setting, whereas a PSADT > 12 months shifts probability toward local failure without excluding metastasis [145,146,147,148]. After LDR-BT, a valid PSADT requires excluding the PSA-bounce window, since a bounce transiently shortens apparent doubling time. When BCR is confirmed, re-staging should employ prostate-specific membrane antigen positron emission tomography/computed tomography (PSMA-PET/CT), which detects recurrence at substantially lower PSA thresholds than those for conventional CT and bone scanning and is guideline-preferred for localizing post-radiotherapy failure; PSADT then informs whether and when to re-stage and to initiate systemic therapy. Additionally, a meta-analysis of 37 studies involving 8409 patients demonstrated that ^68^Ga-PSMA-11 PET/CT and ^68^Ga-PSMA-11 PET/magnetic resonance imaging offer equivalent diagnostic positivity rates for biochemically recurrent prostate cancer (70% vs. 71%, *p* = 0.58) [149]. PSMA-PET/CT re-staging offers a balance of diagnostic sensitivity and cost-effectiveness for localizing post-treatment biochemical relapse.

These observations reinforce that a single static BCR definition cannot serve the full post-treatment course. Because benign bounces cluster in a defined early window and resolve over time, definition performance is time-dependent, and a time-adapted approach would be more optimal, i.e., a high, specificity-favouring ceiling (Phoenix or higher) during the peak bounce window; a transition to a lower nadir-plus-threshold as bounces subside to regain sensitivity; and, in late follow-up, a shift toward a hybrid definition of absolute nadir, paired with consecutive-rises for non-responders, with testosterone monitoring, since residual benign tissue and later hyperplasia make a fixed low absolute cutoff unreliable. Consecutive-rise criteria require standardized sampling intervals to avoid frequency-dependent bias. To date, the literature predominantly supports a 4-year transition point between the early and late follow-up window. At 4 years post-LDR-BT, benign PSA fluctuations have overwhelmingly resolved. Switching from the Phoenix criteria (nadir + 2.0 ng/mL) to a lower absolute threshold (PSA > 0.2 ng/mL) after 4 years allows early local/distant failure detection via PSMA-PET/CT. However, for patients exhibiting extended nadir kinetics or suspected late bounce dynamics, clinicians could conservatively extend the Phoenix evaluation window out to 5 years. Advanced statistical and machine-learning models may ultimately optimize the pairing of each definition with its ideal temporal window.

The operating characteristics of the candidate definitions clarify the trade-off the proposed framework is intended to manage. In a direct LDR-BT comparison, both the Phoenix and surgical definitions achieved 100% specificity for cure at 4 years, with similar accuracy (94.8% vs. 96.3%), but the surgical definition’s negative predictive value for cure was markedly lower (7.7% vs. 29%), meaning that failure to reach PSA ≤ 0.2 ng/mL is a weak indicator of true failure and should not by itself prompt salvage [132]. Conversely, the Phoenix nadir + 2 threshold under-calls slowly rising failures, with nadir PSA and 60-month PSA being the strongest predictors of ultimate failure (odds ratios ~ 20) [133]. Adding a kinetic qualifier to Phoenix reduced its ~ 30% false-positive rate to 6.4% without loss of sensitivity [140], and a high-ceiling consecutive-rise definition (J-POPS) reduced spurious calls substantially—91.1% of Phoenix ‘failures’ later regressed vs. 22.2% of J-POPS failures—at the cost of later detection of genuine recurrence [143]. The time-windowed proposal applies the high-specificity criterion during the bounce-prone early window (favouring fewer false positives when benign bounces are prevalent) and a sensitivity-favouring absolute threshold later (favouring earlier true-recurrence detection once bounces have resolved). Its false-positive and false-negative consequences have not been prospectively quantified; the figures above derive from the component definitions applied separately, not from the combined framework, which remains to be validated.

PSA elevations have to be confirmed on repeat testing, before being classified as recurrence or triggering restaging. Serial values should be measured in the same laboratory on the same platform and reported to at least two decimal places, with paired measurements when the platform changes. At nadirs of 0.1–0.2 ng/mL, a 0.2 ng/mL increment lies only modestly above combined analytical and biological variation (Section 3.2.1), so inter-assay differences alone can generate false calls. Standardized sampling schedules (e.g., quarterly in years 1–3, semi-annually in years 4–5, and annually thereafter) may be applied to eliminate frequency-dependent detection bias. ADT exposure, cessation date and serum testosterone should be documented. During ADT and until testosterone recovers, PSA reflects pharmacologic suppression and rebound; therefore, hormonal status is essential prior to interpreting any post-treatment PSA rise. The late (post 4 years) absolute nadir criterion may only be applied with eugonadal testosterone levels.

**Figure 1:**
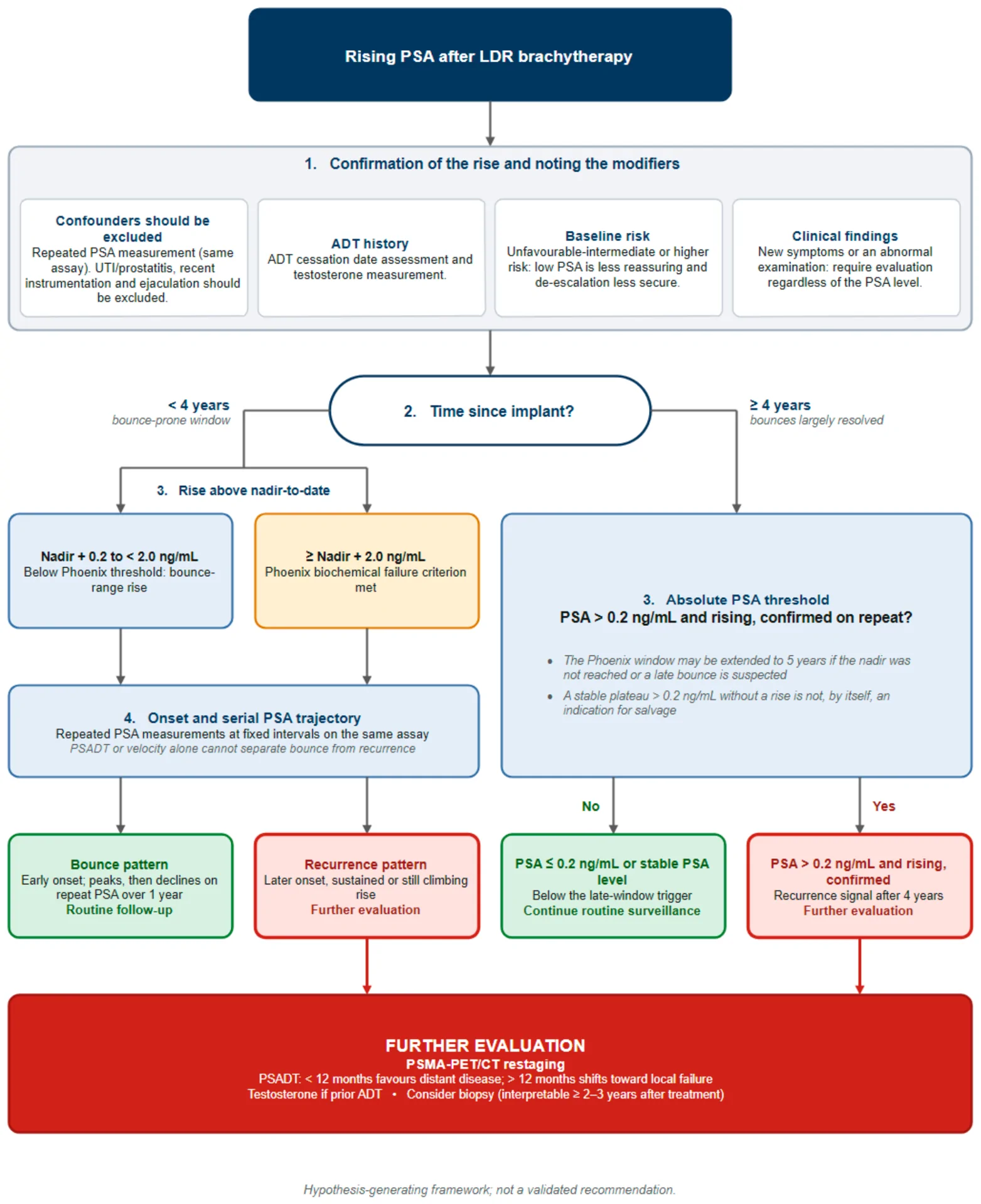
Hypothesis-generating decision pathway for interpreting a rising PSA after LDR brachytherapy. ADT, androgen deprivation therapy; LDR, low-dose-rate; PSA, prostate-specific antigen; PSADT, PSA doubling time; PSMA-PET/CT, prostate-specific membrane antigen positron emission tomography/computed tomography; UTI, urinary tract infection. Nadir-to-date is the lowest PSA recorded before the rise; Phoenix refers to the nadir + 2.0 ng/mL criterion. The pathway is not a validated recommendation and requires prospective validation. Thresholds and time windows are derived from the data discussed in Section 3.

The discussed time-windowed framework is explicitly hypothesis-generating and is not proposed for clinical adoption in advance of prospective validation against clinical endpoints. A lower absolute threshold applied in late follow-up would identify additional patients for investigation but could increase unnecessary PSMA-PET/CT imaging, biopsy, patient anxiety, and salvage intervention in men who are, in fact, cured; conversely, retaining a higher nadir-referenced ceiling could delay detection of genuine recurrence and salvage. The balance of these harms is population- and risk-group-dependent and cannot be resolved from the retrospective evidence synthesized here. To illustrate how clinicians might interpret a rising PSA after treatment, we present a hypothesis-generating algorithm in Figure 1.

### 3.4. Limitations

Several limitations qualify this synthesis. It is a narrative review: no protocol was registered; no formal risk-of-bias instrument was applied; and no quantitative pooling, meta-regression, or certainty grading was performed—all effect estimates are reported descriptively as across-study ranges. The underlying evidence is predominantly retrospective and from a single-institution, with heterogeneous follow-up duration, non-uniform and often undocumented PSA sampling schedules, and variable androgen-deprivation exposure. Bounce and biochemical-recurrence definitions differ across studies, directly affecting reported incidence, timing, duration, and the comparative performance of definitions. Cross-modality comparisons of time to nadir, nadir depth, bounce incidence and amplitude rely on unadjusted or incompletely adjusted between-study comparisons. Reliance on citation chaining for 90 of 143 publications (63%) may over-represent frequently cited work, and restriction to English-language publications introduces language bias. Publication bias cannot be excluded. Finally, the proposed time-adapted framework is derived from retrospective, mostly ^125^I-monotherapy data and has not been prospectively validated against clinical endpoints.

## 4. Conclusions

Two categories of statement should be distinguished. The following are well established: PSA declines over approximately 4–5 years after LDR-BT to a nadir below the surgical threshold in most patients; nadir depth (<0.1–0.5 ng/mL) is a robust prognostic marker, whereas the early decline rate is not; PSA bounce occurs in roughly 30–50% of patients, is a favourable prognostic sign, and frequently mimics recurrence; PSA velocity and doubling time cannot reliably separate benign bounce from true failure, leaving timing of onset as the most useful single discriminator; and younger age and treatment intensity shape bounce behaviour and nadir depth. By contrast, the time-adapted surveillance proposal advanced here—a nadir-referenced (Phoenix) criterion during the early bounce-prone window transitioning to an absolute low-PSA threshold thereafter—is a hypothesis generated from these observations, not a validated recommendation. The available retrospective, definition-heterogeneous evidence does not support adopting a new time-dependent biochemical-recurrence definition for clinical use. Prospective validation against clinical endpoints (disease-specific survival, salvage efficacy), ideally incorporating standardized sampling intervals, testosterone indexing, and modern modelling, is the necessary next step.

## Figures and Tables

**Table 1:** Direction of association between clinical-, patient-, and treatment-related factors and post-treatment PSA dynamic endpoints after definitive radiation therapy for prostate cancer. Arrows give the predominant direction of the effect of each factor on each endpoint relative to the comparator stated in the row label, as summarized in Section 3.1 and Section 3.2. Supporting references are listed in the last column.

Predictor (vs. Comparator)	Time to Nadir	Absolute Nadir	Bounce Incidence	Time to Bounce Onset	Bounce Duration	Bounce Magnitude	Key References
Younger patient age	↑ *	↑ *	↑↑	↓ *	↑ *	↑	[9,11,19,65,103,104,106,107,116,118]
African American vs. Caucasian ancestry	N/E	N/E	↑ *	NS	N/E	N/E	[123]
Larger prostate volume	↑ *	N/E	NS	N/E	N/E	N/E	[11,12,24,87,103,107,121]
Higher pre-treatment tumour burden (iPSA, T stage, GS, risk group)	NS	↑	NS	↑ *	N/E	N/E	[9,11,12,18,19,22,24,28,32,35,39,98,104,105,107,108,119,120,122]
Androgen deprivation therapy (ADT)	↓ *	↓	NS	↓ *	↑ *	NS	[9,26,27,96,103,108,110,126]
Higher radiation dose/better dosimetric coverage (BED, D90, V100)	↑ *	↓	NS	N/E	NS	N/E	[24,31,32,33,34,45,48,80,82,104]
^103^Pd vs. ^125^I (LDR-BT)	↓ *	N/E	↓ *	↓ †	↓ †	N/E	[43,112]
High-dose-per-fraction radiation therapy (HDR-BT, SBRT, H-EBRT) vs. CF-EBRT	↑	↓	↑	↓	↑ *	↑	[9,17,42,45,89,105,117,122,127,128]

Symbols: ↑, the factor increases the endpoint (longer interval, higher value, or higher frequency); ↓, the factor decreases the endpoint (shorter interval, lower value, or lower frequency); ↑↑, strong and consistent increase; NS, evaluated, but no significant or consistent association, including associations lost via multivariable adjustment; N/E, not evaluated or not reported in the reviewed literature; *, based on isolated, limited, or conflicting evidence (see text); †, observed only in patients receiving ADT.

## Data Availability

No new data were created or analysed in this study. Data sharing is not applicable to this article.

## Supplementary Materials

The following supporting information can be downloaded at: https://www.mdpi.com/article/10.3390/diagnostics16213689/s1, File S1: Literature Search Strategy. Table S1: Comparative summary of included clinical studies. Table S2: PSA bounce study-level summary in low-sose rate prostate brachytherapy for the nadir + 0.2 ng/mL definition of bounce.

## Abbreviations

The following abbreviations are used in this manuscript:

ADT	Androgen deprivation therapy
ASTRO	American Society for Therapeutic Radiology and Oncology
BCR	Biochemical recurrence
BED	Biologically effective dose
bRFS	Biochemical recurrence-free survival
BT	Brachytherapy
CAPRA	Cancer of the Prostate Risk Assessment
CF-EBRT	Conventionally fractionated external beam radiotherapy
D90	Minimum dose received by 90% of the prostate volume
DFS	Disease-free survival
EBRT	External beam radiotherapy
GS	Gleason score
Gy	Gray
Gy_2_	Biologically effective dose in gray, calculated using an α/β ratio of 2 Gy
H-EBRT	Hypofractionated external beam radiotherapy
IMRT	Intensity-modulated radiotherapy
iPSA	Initial (pretreatment/baseline) prostate-specific antigen
J-POPS	Japanese Prostate Cancer Outcome Study
LDR	Low-dose-rate
nPSA	Prostate-specific antigen nadir
nPSA12	Prostate-specific antigen nadir within 12 months
PC	Prostate cancer
PSA	Prostate-specific antigen
PSADT	Prostate-specific antigen doubling time
PSMA-PET/CT	Prostate-specific membrane antigen positron emission tomography/computed tomography
RP	Radical prostatectomy
RT	Radiotherapy
SBRT	Stereotactic body radiotherapy
V100/V150/V200	Volume receiving 100%, 150% or 200% of the prescription dose
