# Supplementary material for: PSA Dynamics, Bounce, and Biochemical Recurrence Definitions After Low-Dose-Rate Brachytherapy for Prostate Cancer: A Narrative Review with Cross-Modality Comparison
Source: Diagnostics (Basel). 2026 Nov 8;16(21):3689. doi: 10.3390/diagnostics16213689 (PMC13763180; doi:10.3390/diagnostics16213689)
Supplement: Supplementary file 1 [file diagnostics-16-03689-s001.zip › File S1.pdf]

File S1: Literature search strategy. Detailed database search queries, boolean operators, and explicit search strings applied across PubMed, Scopus, and Web of Science.

A single conceptual Boolean strategy with four concept blocks (disease, risk group, intervention, biomarker) was translated into the native syntax of each platform. Searches were restricted to title, abstract, and keyword fields and to publications dated January 1, 2010 to March 3, 2026. No language filter was applied at the search stage; non-English records were excluded during screening. No controlled vocabulary (MeSH, Emtree) was used. The strategy was not peer-reviewed (PRESS) before execution.

Concept blocks (platform-independent)

**Block**	**Concept**	**Terms**
1	Disease	"prostate cancer"
2	Risk group	"low risk" OR "intermediate risk" OR "favourable risk" OR "unfavourable intermediate risk"
3	Intervention	"LDR brachytherapy" OR "low dose rate brachytherapy" OR "low-dose-rate brachytherapy" OR "LDR-BT" OR "LDR BT" OR "seed implantation" OR "permanent seed implant"
4	Biomarker	"PSA" OR "prostate-specific antigen"
—	Combination	1 AND 2 AND 3 AND 4

PubMed (MEDLINE) search string:

Line-by-line strategy:

```
#1  "prostate cancer"[tiab]
#2  "low risk"[tiab] OR "intermediate risk"[tiab] OR
"favourable risk"[tiab] OR "unfavourable intermediate
risk"[tiab]
#3  "LDR brachytherapy"[tiab] OR "low dose rate
brachytherapy"[tiab] OR "low-dose-rate brachytherapy"[tiab]
OR "LDR-BT"[tiab] OR "LDR BT"[tiab] OR "seed
implantation"[tiab] OR "permanent seed implant"[tiab]
#4  "PSA"[tiab] OR "prostate-specific antigen"[tiab]
#5  #1 AND #2 AND #3 AND #4
#6  #5 AND ("2010/01/01"[dp] : "2026/06/30"[dp])
```

Single-line equivalent:

```
("prostate cancer"[tiab]) AND ("low risk"[tiab] OR
"intermediate risk"[tiab] OR "favourable risk"[tiab] OR
"unfavourable intermediate risk"[tiab]) AND ("LDR
brachytherapy"[tiab] OR "low dose rate brachytherapy"[tiab]
OR "low-dose-rate brachytherapy"[tiab] OR "LDR-BT"[tiab] OR
"LDR BT"[tiab] OR "seed implantation"[tiab] OR "permanent
seed implant"[tiab]) AND ("PSA"[tiab] OR "prostate-specific
antigen"[tiab]) AND ("2010/01/01"[dp] : "2026/06/30"[dp])
```

Syntax notes: [tiab] searches title, abstract, and author-supplied keywords in current PubMed. Quoted phrases with a field tag turn off Automatic Term Mapping, so no MeSH explosion occurred. [dp] = date of publication.

Scopus search string:

```
TITLE-ABS-KEY ( "prostate cancer" )
AND TITLE-ABS-KEY ( "low risk" OR "intermediate risk" OR
"favourable risk" OR "unfavourable intermediate risk" )
AND TITLE-ABS-KEY ( "LDR brachytherapy" OR "low dose rate
brachytherapy" OR "low-dose-rate brachytherapy" OR "LDR-BT"
OR "LDR BT" OR "seed implantation" OR "permanent seed
implant" )
AND TITLE-ABS-KEY ( "PSA" OR "prostate-specific antigen" )
AND PUBYEAR > 2009 AND PUBYEAR < 2027
```

Syntax notes: TITLE-ABS-KEY searches title, abstract, author keywords, and indexed keywords. Double quotes run a "loose" phrase search, which ignores punctuation (so "LDR-BT" and "LDR BT" are equivalent) and matches plural forms.

Web of Science Core Collection search string:

```
TS=("prostate cancer")
AND TS=("low risk" OR "intermediate risk" OR "favourable
risk" OR "unfavourable intermediate risk")
AND TS=("LDR brachytherapy" OR "low dose rate
brachytherapy" OR "low-dose-rate brachytherapy" OR "LDR-BT"
OR "LDR BT" OR "seed implantation" OR "permanent seed
implant")
AND TS=("PSA" OR "prostate-specific antigen")
AND PY=(2010-2026)
```

Syntax notes: TS= (Topic) searches title, abstract, author keywords, and KeyWords Plus.

Supplementary search methods (citation chaining)

Item	Description

Method	Manual backward citation chaining (reference-list screening) of the 53 included database-derived articles
Iterations	Repeated until conceptual saturation
Target content	Foundational and pre-2010 studies; cross-modality comparisons; high-risk and mixed-risk cohorts; BCR-definition studies
Eligibility	Same exclusion criteria as the database arm (non-English, single-case reports, cohorts < 20 patients, no relevant PSA-kinetic data, substantial data-quality limitations)
Records added	90
